# First-in-human study assessing safety, tolerability, and pharmacokinetics of 2-hydroxybenzylamine acetate, a selective dicarbonyl electrophile scavenger, in healthy volunteers

**DOI:** 10.1186/s40360-018-0281-7

**Published:** 2019-01-05

**Authors:** Lisa M. Pitchford, John A. Rathmacher, John C. Fuller, J. Scott Daniels, Ryan D. Morrison, Wendall S. Akers, Naji N. Abumrad, Venkataraman Amarnath, Patricia M. Currey, L. Jackson Roberts, John A. Oates, Olivier Boutaud

**Affiliations:** 1 Metabolic Technologies, Inc., Ames, IA 50010 USA; 2 Department of Animal Science, Iowa State University, Ames, IA 50010 USA; 3 Sano Informed Prescribing Inc., Franklin, TN 37067 USA; 4 Department of Pharmaceutical Sciences, Lipscomb University College of Pharmacy, Nashville, TN 37204 USA; 5 Department of Pharmacology, Division of Clinical Pharmacology, Vanderbilt University, Nashville, TN 37232 USA; 6 Department of Surgery, Vanderbilt University Medical Center, Nashville, TN 37232 USA; 7 Department of Medicine, Division of Clinical Pharmacology, Vanderbilt University Medical Center, Nashville, TN 37232 USA; 8 Department of Medicine, Division of Clinical Pharmacology, Vanderbilt University Medical Center, Nashville, TN 37232 USA

**Keywords:** Safety, Pharmacokinetics, Humans, Salicylamine, γ-Ketoaldehydes

## Abstract

**Background:**

2-Hydroxybenzylamine (2-HOBA) is a selective scavenger of dicarbonyl electrophiles that protects proteins and lipids from being modified by these electrophiles. It is currently being developed for use as a nutritional supplement to help maintain good health and protect against the development of conditions associated with dicarbonyl electrophile formation, such as the cognitive decline associated with Mild Cognitive Impairment and Alzheimer’s disease.

**Methods:**

In this first-in-human study, the safety, tolerability, and pharmacokinetics of six ascending single oral doses of 2-HOBA acetate were tested in eighteen healthy human volunteers.

**Results:**

Reported adverse events were mild and considered unlikely to be related to 2-HOBA. There were no clinically significant changes in vital signs, ECG recordings, or clinical laboratory parameters. 2-HOBA was fairly rapidly absorbed, with a t_max_ of 1–2 h, and eliminated, with a t_1/2_ of approximately 2 h. Both t_max_ and t_1/2_ were independent of dose level, while C_max_ and AUC increased proportionally with dose level.

**Conclusions:**

2-HOBA acetate was safe and well-tolerated at doses up to 825 mg in healthy human volunteers, positioning it as a good candidate for continued development as a nutritional supplement.

**Trial registration:**

This study is registered at ClinicalTrials.gov (NCT03176940).

**Electronic supplementary material:**

The online version of this article (10.1186/s40360-018-0281-7) contains supplementary material, which is available to authorized users.

## Background

Inflammation and oxidative stress, which have been implicated as potential mediators in the development and progression of many conditions, result in the formation of extremely reactive dicarbonyl electrophiles. These dicarbonyls react with lysine residues to form protein adducts that can alter the function and interactions of various cellular proteins [1, 2]. Proteins adducted by one such highly reactive dicarbonyl, isolevuglandin, are found at elevated levels in a number of health conditions, including Alzheimer’s disease [3], atherosclerosis [4], hypertension [5], and liver disease [6]. Dicarbonyl electrophiles also have been mechanistically associated with the development of Alzheimer’s disease, as they have been shown to enhance the oligomerization and neurotoxicity of amyloid beta [7, 8].

Fortunately, dicarbonyl electrophiles can be specifically neutralized by a novel class of scavenger molecules [9]. One of these scavenger molecules, 2-hydroxybenzylamine (2-HOBA), reacts substantially faster with these dicarbonyls than does lysine, preventing dicarbonly-associated protein modifications. 2-HOBA, which is naturally found in buckwheat [10], is orally available [11] and crosses the blood brain barrier, resulting in brain 2-HOBA levels approximately twice as high as plasma levels [11]. Administration of 2-HOBA prevented the age-associated working memory deficit in the hApoE4 mouse model [12], suggesting this inhibition of dicarbonyl protein modification could help maintain hippocampal function.

A series of preclinical safety studies, including acute and short-term rodent studies [13] as well as in vitro safety pharmacology studies [14] and sub-chronic rat [15] and rabbit studies [16] indicated no toxicity concerns of 2-HOBA acetate within the therapeutic dose range. Thus, the objective of the present investigation was to perform the initial evaluation of 2-HOBA acetate in humans. This first-in-human study assessed the safety, tolerability, and pharmacokinetics of single ascending doses of 2-HOBA acetate in healthy humans.

## Methods

This study is registered at ClinicalTrials.gov (NCT03176940). The study protocol was approved by the Vanderbilt University Institutional Review Board. All participants provided written informed consent before participating in the study.

### Subjects

Healthy male and non-pregnant female volunteers over 18 years of age were eligible to participate. Subjects were not permitted to take any medications 2 weeks prior to or during the study. Exclusion criteria included known cardiac, kidney, or hepatic disease; presence of diseases that could manifest morbidity or symptoms/signs that could confound interpretation of the study results; the need to discontinue any drug administered as standard of care treatment; and the unwillingness or inability to use approved birth-control methods.

### Compound

2-HOBA (as the acetate salt, CAS 1206675–01-5) was obtained from TSI (China) Co., Ltd. (Shanghai, China). A commercial production lot was used (Lot 16120312). Our laboratory verified the purity of the commercial lot to be > 99% via HPLC and NMR spectroscopy. Hard gel capsules (Capsugel, Jiangsu, China) containing 50, 110, and 412.5 mg of 2-HOBA acetate (corresponding to 34, 75, and 281 mg 2-HOBA) were prepared by TSI (China) Co., Ltd. Determinations of average fill weight, uniformity of weight, disintegration, 2-HOBA content, acetate content, and microbial and analytical tests were within all specification limits.

### Study design

This study was an open-label, single ascending dose study designed to assess pharmacokinetics, safety, and tolerability of single doses of 2-HOBA acetate. A 3 + 3 clinical trial design with a modified Fibonacci sequence dosing scheme [17] was used with a starting dose of 50 mg; thereafter, dosages were increased to 100, 200, 330, 550, and 825 mg. These doses of 2-HOBA acetate correspond to 34, 68, 136, 224, 373, and 560 mg 2-HOBA. Each dose escalation was initiated only after reviewing safety data from all subjects receiving the previous dose.

Subjects were admitted to the Vanderbilt University Clinical Research Center and remained on the unit for 24 h after administering 2-HOBA acetate orally in capsules to participants. Though this study did not include a placebo control, staff nurses and participants were blinded to the capsule dosage content. Subjects were monitored at protocol-defined intervals for 24 h after administration of 2-HOBA. Safety assessments included vital signs (heart rate, respiration rate, blood pressure, and SpO_2_), clinical laboratory parameters (blood biochemistry, hematology, and urinalysis), 12-lead ECGs, and potential adverse event assessments. All adverse events were recorded, regardless of whether they were considered to be study-related.

### Pharmacokinetic sampling and analysis

Blood samples for pharmacokinetic analyses were collected at baseline, 0.5, 1, 2, 4, 8, and 24 h after 2-HOBA acetate administration for all dose levels. A 0.25-h sample was only collected for dosages ≤200 mg, and a 6-h sample was only collected for dosages ≥330 mg. Plasma concentrations of 2-HOBA as well as the primary metabolite of 2-HOBA, salicylic acid, were determined for each time point.

[^2^H_4_]-2-HOBA, prepared by Dr. Venkataraman Amarnath as previously described [11], was used as an internal standard. An internal standard solution (100 ng/mL) of ([^2^H_4_]-2-HOBA was prepared in acetonitrile and added to all standards, quality control samples, and patient samples. Standard and quality control samples of 1 mg/mL 2-HOBA were prepared in water. Eight standard curve samples (5, 10, 20, 100, 200, 1000, 2000, and 5000 ng/mL) were prepared with blank human plasma (Bioreclamation, Westbury, NY). In addition, three quality control samples (15, 300, and 3000 ng/mL) were prepared in blank human plasma. Plasma samples were allowed to thaw at room temperature and then vortexed. Internal standard solution (400 μL) and 100 μL of either plasma, quality controls, or standards were added and mixed in a protein precipitation filter 96-well plate (Phenomenex, Torrance, CA). The solution was eluted into a 96-well plate using a positive pressure manifold and then dried under nitrogen gas at 40 °C. The samples were then reconstituted in 97/3 *v*/v water/acetonitrile with 10 mM ammonium formate for analysis. Liquid chromatography tandem mass spectrometry analysis of 2-HOBA was performed with Shimadzu Nexera X2 LC-30 AD pumps, column oven, and degasser (Kyoto, Japan) (column: C18 2.1 × 50 mm, 1.7 μm, Phenomenex, Torrance, CA) coupled with a Sciex QTrap 5500 mass spectrometer with TurboV ion source (Framingham, MA). Quantification of 2-HOBA was performed using electrospray ionization in positive ionization mode. The column temperature was set to 60 °C and the flow rate was 0.5 mL/min. A gradient of 3–90 %B from 0 to 0.90 min was established by using a mobile phase A of 10 mM ammonium formate in water and mobile phase B of 1% formic acid in acetonitrile. Quantification of 2-HOBA was validated over the range of 5–5000 ng/mL, with within-run precision of 3.7–7.0%, bias − 9.7 – 2.8 and between run precision of 4.4–6.2%, bias − 7.1 – 1.64. All standards and quality control samples met acceptance criteria (standard curve R^2^ > 0.90, 66.7% of all QC samples and at least 50% at each concentration within 15% of nominal concentration).

Plasma concentration-time data was imported into Phoenix WinNonlin® 8.0 software (Certara USA, Inc., Princeton, NJ) to estimate the oral pharmacokinetic parameters of 2-HOBA from individual subjects at each dose level. Non-compartmental analysis using Model 200 (Plasma; Single Extravascular Dose; Linear Log Trapezoidal Method) was performed on each plasma concentration-time profile to estimate individual pharmacokinetic parameters – half-life, area under the concentration-time curve (AUC), maximum observed plasma concentration (Cmax), and the time to reach the maximum observed plasma concentration (Tmax).

### Statistical analyses

Descriptive statistics (means, standard deviations, standard error) were used for demographics, safety, and pharmacokinetic assessments.

## Results

### Study population

A total of 18 volunteers were enrolled in and successfully completed the study (3 subjects at each dose level). Subject demographics are provided in Table 1 and were similar across dose groups.

**Table 1:** Demographic Characteristics

	2-Hydroxybenzylamine acetate dose						
	50 mg	100 mg	200 mg	330 mg	550 mg	825 mg	Total
Volunteers (*n*)	3	3	3	3	3	3	18
Sex: female [*n* (%)]	2 (66.7)	1 (33.3)	0 (0)	3 (100)	1 (33.3)	2 (66.7)	9 (50.0)
Age (y)	25.7 ± 2.1	32.7 ± 6.4	27.7 ± 5.7	26.3 ± 4.0	28.0 ± 6.0	23.0 ± 2.6	27.2 ± 5.0
Height (cm)	174.3 ± 7.6	184.6 ± 11.7	174.2 ± 4.0	165.7 ± 6.8	175.7 ± 17.8	160.4 ± 5.6	172.5 ± 11.6
Weight (kg)	60.7 ± 2.1	95.0 ± 37.0	83.3 ± 30.6	60.0 ± 8.0	87.7 ± 33.8	68.3 ± 14.2	75.8 ± 25.1
BMI (kg/m^2^)	20.1 ± 2.3	27.3 ± 7.8	27.2 ± 8.8	21.8 ± 1.3	27.5 ± 5.0	26.4 ± 3.8	25.0 ± 5.6
Race							
Hawaiian/Pacific Islander	0 (0)	0 (0)	0 (0)	0 (0)	1 (33.3)	0 (0)	1 (5.5)
White	3 (100)	3 (100)	3 (100)	3 (100)	2 (66.7)	3 (100)	17 (94.4)
Ethnicity							
Hispanic/Latino	0 (0)	0 (0)	0 (0)	0 (0)	1 (33.3)	0 (0)	1 (5.5)
Not Hispanic/Latino	3 (100)	3 (100)	3 (100)	3 (100)	2 (66.7)	3 (100)	17 (94.4)

Data are presented as means ± SD unless otherwise noted

### Safety

All reported adverse events are summarized in Table 2. Five participants (28%) reported at least 1 adverse event during the study. The most common reported adverse event (2 incidences) was frequent urination (2 subjects, 11%). All adverse events were mild in intensity. No adverse events were determined to be study-related, and there was no dose-dependent increase in adverse event frequency or severity. No clinically significant changes in ECG recordings, vital signs, or laboratory parameters that were considered to be related to 2-HOBA were observed. There were no serious adverse events or deaths.

**Table 2:** Summary of reported adverse events by dose

	2-Hydroxybenzylamine acetate dose						Total (*n* = 18)
	50 mg (*n* = 3)	100 mg (*n* = 3)	200 mg (*n* = 3)	330 mg (*n* = 3)	550 mg (*n* = 3)	825 mg (*n* = 3)	
Any event, n (%)	3 (100)	0	1 (33)	1 (33)	0	0	5 (28)
Frequent urination	2 (67)	0	0	0	0	0	2 (11)
Headache	0	0	1 (33)	0	0	0	1 (5.5)
Itchy throat	1 (33)	0	0	0	0	0	1 (5.5)
Rash	1 (33)	0	0	0	0	0	1 (5.5)
Sleepiness	1 (33)	0	0	0	0	0	1 (5.5)
Abdominal bloating	0	0	0	1 (33)	0	0	1 (5.5)

### Pharmacokinetics

**Fig. 1:**
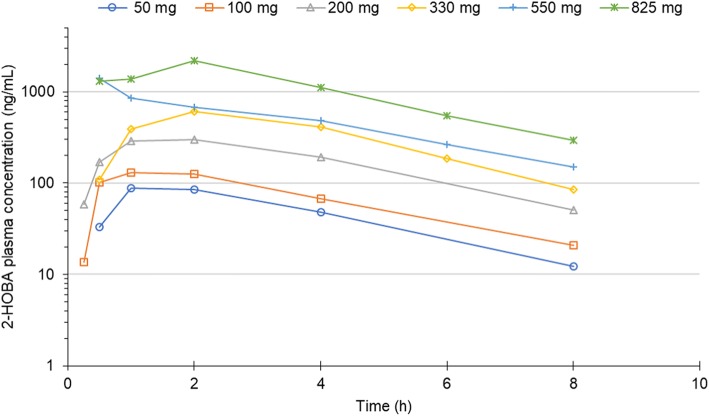
2-Hydroxybenzylamine (2-HOBA) plasma concentrations after oral administration of 2-HOBA acetate. 2-HOBA plasma concentration was measured for 24 h after oral administration of six ascending single doses of 2-HOBA acetate in healthy subjects (n=3 per dose level). Blood samples for pharmacokinetic analyses were collected at baseline, 0.5, 1, 2, 4, 8, and 24 h after 2-HOBA acetate administration for all dose levels. A 0.25-hour sample was only collected for dosages ≤ 200 mg, and a 6-h sample was only collected for dosages ≥ 330 mg. No 2-HOBA was detectable prior to administration (time 0) or at 24 h post-administration (assay limit of detection = 5 ng/mL)

Mean 2-HOBA plasma concentration-time profiles and pharmacokinetic parameter estimates are shown in Fig. 1 and Table 3, respectively. Following oral administration of single doses of 2-HOBA, dose-dependent changes were observed for maximal plasma concentration (C_max_) and area under the concentration-time curve (AUC). The mean time to reach C_max_ was 1.6 h and the mean half-life of 2-HOBA was 2.1 h.

**Table 3:** Mean 2-hydroxybenzylamine pharmacokinetic parameters after a single oral dose

Parameter	2-Hydroxybenzylamine acetate dose					
	50 mg (*n* = 3)	100 mg (*n* = 3)	200 mg (*n* = 3)	330 mg (*n* = 3)	550 mg (*n* = 3)	825 mg (*n* = 3)
Half-life (h)	2.04	2.33	2.03	1.77	2.32	2.13
C_max_ (ng/mL)	90	156	369	669	1636	2510
t_max_ (h)	1.33	1.33	1.83	1.67	1.67	1.67
AUC (h·ng/mL)	396	622	1490	2690	4417	9053
AUC_extrap_ (%)	9.1	11.2	10.0	8.2	11.2	10.3

*C*_*max*_ maximum observed plasma concentration, *t*_*max*_ time to reach, *C*_*max*_ AUC, area under the concentration-time curve from zero to infinity, *AUC*_*extrap*_ percentage of the AUC extrapolated from the last observed time point

Clearance and volume of distribution are not reported due to the unknown value of F (bioavailability)

Plasma concentrations of the primary metabolite of 2-HOBA, salicylic acid were also measured. Systemic exposure to salicylic acid following oral administration of single doses of 2-HOBA acetate at each dose level is shown in Additional file 1: Figure S1 and quantified in Additional file 1: Table S1, both located in Additional file 1. Following oral administration of 2-HOBA, dose-dependent changes were observed in the systemic exposure (C_max_ and AUC) of salicylic acid. The t_max_ for salicylic acid ranged from 2.67 to 4.67 h and tended to increase as the 2-HOBA dose increased.

## Discussion

The results of this first-in-human study demonstrate that single doses of 2-HOBA acetate up to 825 mg were well tolerated by healthy individuals. No serious adverse events or dose-limiting adverse events were observed. All reported adverse events were mild in intensity and unlikely to be 2-HOBA-related. There were no clinically significant findings in vital signs, ECG recordings, or clinical laboratory parameters.

After oral administration of a single dose, 2-HOBA was readily absorbed, with a t_max_ of 1 to 2 h after administration at all dose levels. Systemic exposure to 2-HOBA was dose-dependent, as was the exposure of salicylic acid, the primary metabolite of 2-HOBA. The pharmacokinetic values for salicylic acid observed in this study were similar to those observed after single doses of 40.5–324 mg of acetylsalicylic acid (aspirin) [18], which is rapidly hydrolyzed to salicylic acid after oral administration. This suggests the tested dose range of 2-HOBA acetate induces similar salicylic acid exposure to that observed with low-to-moderate doses of acetylsalicylic acid.

The terminal slopes of the log-linear phase of the plasma-concentration time profiles for 2-HOBA were parallel and independent of dose, which is reflected in the similar half-life at each dose level. 2-HOBA appears to have a relatively short half-life (~ 2 h). These pharmacokinetic properties of 2-HOBA in humans are generally similar to those previously observed in mice [11]. Clearance and volume of distribution are not reported due to the unknown value of F (bioavailability) following oral administration of 2-HOBA acetate.

These observations supporting that orally administered 2-HOBA acetate is well-tolerated and safe in humans comprise an important addition to our portfolio of preclinical 2-HOBA safety data. The good safety profile to date combined with the preclinical efficacy established in mice at risk for age-related cognitive decline support continued development of 2-HOBA as a nutritional supplement to enhance cognitive health and support healthy brain aging. Additionally, although our development of 2-HOBA has been primarily focused on the cognitive decline associated with Mild Cognitive Impairment or Alzheimer’s disease, there is also evidence for beneficial effects of 2-HOBA on other aspects of neurological health associated with the accumulation of dicarbonyl adducts, such as seizure-associated cognitive dysfunction. 2-HOBA attenuated neuronal loss, astrogliosis, and spatial and reference memory deficits in mouse models of epilepsy without affecting epileptic injury [19].

There is also evidence for beneficial effects of 2-HOBA on cardiovascular health, most notably blood pressure. 2-HOBA attenuated angiotensin-II induced hypertension and renal damage in mice [5], normalized blood pressure in a murine model of hypertension [20], and prevented the development of pulmonary arterial hypertension in mice with mitochondrial oxidant injury [21]. Exposure to 2-HOBA also attenuated molecular aging and extended longevity in *C. elegans*, increasing lifespan by more than 50% [22]. Together, these additional preclinical studies support the potential for 2-HOBA to have wide-ranging protective effects against oxidant-related disease and dysfunction.

A limitation of this first-in-human study is the relatively narrow age range of volunteers. Though older individuals were not excluded from participation in this trial, the enrolled cohort ranged in age from 20 to 40 years; this limits the generalizability of these findings to broader populations. Follow-up studies should specifically target older individuals to identify any unique adverse effects or pharmacokinetic properties in this population.

## Conclusions

In summary, 2-HOBA acetate was safe and well-tolerated in single dose administration up to 825 mg in healthy human volunteers. The pharmacokinetic profile was reasonable, exhibiting fairly rapid absorption and elimination of 2-HOBA. Next steps include evaluating the safety and tolerability of multiple doses of 2-HOBA acetate in healthy individuals as well as older individuals more representative of the population segment at elevated risk for developing Alzheimer’s disease. We will also confirm in humans that 2-HOBA crosses the blood brain barrier, as has been previously demonstrated in rodents [11].

## Additional file

Additional file 1: **Figure S1** and **Table S1.** Mean exposure and pharmacokinetics of the primary metabolite of 2-HOBA, salicylic acid, after oral administration of 2-HOBA acetate. (DOCX 21 kb)

## Abbreviation

2-HOBA: 2-hydroxybenzylamine
