# Supplementary material for: First-in-human study assessing safety, tolerability, and pharmacokinetics of 2-hydroxybenzylamine acetate, a selective dicarbonyl electrophile scavenger, in healthy volunteers
Source: BMC Pharmacol Toxicol. 2019 Jan 5;20:1. doi: 10.1186/s40360-018-0281-7 (PMC6321651; doi:10.1186/s40360-018-0281-7)
Supplement: Supplementary file 1 — Figure S1 and Table S1. Mean exposure and pharmacokinetics of the primary metabolite of 2-HOBA, salicylic acid, after oral administration of 2-HOBA acetate. (DOCX 21 kb) [file 40360_2018_281_MOESM1_ESM.docx]

**Supplemental Figure S1.** Mean plasma concentrations of salicylic acid after oral administration of six ascending single oral doses of 2-hydroxybenzylamine acetate in healthy subjects (*n*=3 per dose level).

| **Supplemental Table S1. Mean exposure of salicylic acid after a single oral dose of 2-hydroxybenzylamine acetate** | | | | | | |
| --- | --- | --- | --- | --- | --- | --- |
| Parameter | 2-Hydroxybenzylamine acetate dose | | | | | |
|  | 50 mg (*n*=3) | 100 mg (*n*=3) | 200 mg (*n*=3) | 330 mg (*n*=3) | 550 mg (*n*=3) | 825 mg (*n*=3) |
| C_max_ (ng/mL) | 1072 | 1765 | 3948 | 8444 | 8611 | 17308 |
| T_max_ (h) | 2.67 | 3.33 | 3.33 | 4.00 | 4.67 | 4.67 |
| AUC_0-inf_ (h⋅ng/mL) | 8238 | 14578 | 34212 | 74402 | 77374 | 161588 |
| AUC_extrap_ (%) | 9.2 | 10.9 | 1.7 | 2.0 | 1.7 | 1.4 |
| C_max_, maximum observed plasma concentration; T_max_, time to reach C_max_; AUC_0-inf_, area under the concentration-time curve from zero to infinity; AUC_extrap_, percentage of the AUC_0-inf_ extrapolated from the last observed time point | | | | | | |
